# The fight for bodily agency, human rights and lived experiences of LGBTIQ+ persons should be celebrated every day

**DOI:** 10.1002/jia2.25911

**Published:** 2022-05-16

**Authors:** Denis Nzioka

**Affiliations:** ^1^ Denis Nzioka News Agency and Service Activist Nairobi Kenya

Globally, there is increased inequality based on health, race, gender, nationality, sexuality, wealth and other tiers of social stratification. Sex and sexuality lie at the heart of these issues, as stubborn cultural and ideological beliefs create and reinforce discrimination. Sexual and gender minority persons face dangerous levels of homophobia, transphobia, gender‐based and political violence, and dislocation from their homes and sometimes from their own countries. In addition, marginalized communities bear the brunt of corrupt governments, and an ever‐shrinking space where they can freely and safely create change.

May 17, the International Day against Homophobia, Transphobia and Biphobia, is a day for sexual and gender minority persons to celebrate this year's theme of “*Our Bodies, Our Lives, Our Rights*,” which speaks to the heart of this chaotic world we live in and is particularly important as we have witnessed reports of bodily and rights violations, loss of lives, and continued shame and indignity that too many of us continue to face.

These three stories highlight why we need to protect their rights, de‐shame their bodies and continue to celebrate their lives.

Eddie Ndopu is a South African‐based disability activist and global advocate for the United Nations’ Sustainability Development Goals [1]. He was a speaker at the Global Black Gay Men Connect's pre‐conference at ICASA 2021, where he talked about the “intersectionality” of disability and being queer. Intersectionality recognizes that there is no one identity or situation of a human being. We have multiple identities, face multiple issues and our lives intersect with multiple forms of oppression. It is thus expected that the more complex and diverse we are, the more we face marginalization and discrimination on multiple levels, based on these identities. He began his remarks by focusing on the word “intersectionality,” which offers us an opportunity to develop new methodologies and new insights on how we tackle exclusion and discrimination.

“It is important that when we speak of ‘leaving no one behind’ within our own community, we do so by paying particular attention to those who embody identities that position us at a profound disadvantage when it comes to issues of autonomy, access to comprehensive sexuality education, and sexual and reproductive health rights,” he said.

He gave an example of how, when people think about accessibility, they often imagine building a ramp to a building. He noted “a ramp facilitates entry into a building, but it doesn't necessarily make a space ‘accessible.’ What makes the space accessible is the freedom, the sense of belonging, the agency and the dignity of that space.”

“It is important we think about accessibility, beyond the built‐in environment in the context of disability justice and think about access to self‐actualisation, bodily autonomy, and access to be able to be regarded as sexual beings. Often disabled people are de‐sexualised and it's a broader manifestation of the dehumanisation we experience on account of able‐ism.”

He challenged everyone to rethink what intersectionality means for those who may be excluded from the common discourse around sexual rights and discrimination.

A 2021 report [2] on the LGBT+ situation in Ukraine found that far‐right groups continued to “purposefully monitor and attack LGBT+ events, organizations and individual activists.”

However, despite opposition from the country's religious and some political leaders, on 19 September 2021, it was estimated that some 7000 people had demonstrated in Kyiv, Ukraine, in the annual March for Equality to show support for the country's LGBTIQ+ community [3].

The last 5 months have been a far cry from the colourful costumes and rainbow flags that characterized the march, following the Russian invasion of Ukraine and a refugee crisis of LGBTIQ+ Ukrainians and those of other nationalities who had found refuge in the country. After the invasion, Ambassador Bathsheba Nell Crocker, the U.S. Representative to the Office of the United Nations and Other International Organizations in Geneva, had given a warning [4] that Russian forces might target gay Ukrainians specifically. “These acts, which in past Russian operations have included targeted killings, kidnappings/forced disappearances, unjust detentions, and the use of torture, would likely target those who oppose Russian actions, including Russian and Belarusian dissidents in exile in Ukraine, journalists and anti‐corruption activists, and vulnerable populations such as religious and ethnic minorities and LGBTQI+ persons,” she said.

This warning highlights the continued government oppression of sexual and gender minorities all over the world, especially in Eastern Europe and Africa, with the rise [5] of populism and neo‐hatred groups. When war further breaks families and livelihoods, it is important to put a spotlight and support LBGTIQ+ persons who face multiple levels of oppression.

Public interest litigation is increasingly being adopted by activists around the world to push for the protection of human rights of LGBTIQ+ persons. This is nowhere clearer than in Africa, where advocates in Ghana [6], Botswana [7], Mozambique [8], Namibia [9], Kenya [10], Angola [11] and Senegal [12] are seeking juridical interpretation of equality laws, demanding broader protections and overhauls of legislation used to oppress their communities.

Colonial‐era laws in most African countries stipulated harsh prison terms, varying from years to a lifetime, for displays of same sex affection and acts. Other violations stipulated in law include forced anal examinations [13] and HIV testing. After decolonization, some of these countries proceeded to introduce subsidiary legislation in a bid to further criminalize and target persons who identify as LGBTIQ+. Organizations that were LGBTIQ+ led or that supported LGBTIQ+ rights were also targeted, which has recently occurred in Uganda [14], Nigeria [15] and Ghana [16].

What is clear is that enactment or even public campaigns around such subsidiary legislation ultimately leads to violence, extortion and, at worst, murder. However, it has been shown that indeed the courts can be a source of justice for not only LGBTIQ+ communities but also communities of women living with HIV who have been sterilized without their consent [17] and other marginalized groups.

Hate is oppressive, especially against sexual and gender minorities. No one country or community is immune from it. Be it in South Africa and Ukraine, or in Asia, Latin America, North Africa and other regions where homo‐ and trans‐phobic laws persist, the right to exist and the need to be loved and appreciated is often not guaranteed. Many LGBTIQ+ persons were lost, and more are living difficult lives in complex environments. Some stories will never be known. Others will be forgotten. Often, examples of resilience and hope may seem small and unassuming, but they do make a difference. It is these small “pockets of change” that add up to the big change that are so desired.

May 17 is a day when all of us can again renew our commitment to stand up for human rights, promote bodily autonomy, agency and expression, and celebrate the diverse lives of the LGBTIQ+ communities.

## COMPETING INTERESTS

None noted.

## AUTHOR'S CONTRIBUTIONS

DN developed and wrote the Viewpoint.

## References

[1] Sustainable Development Goal Advocates . Edward (Eddie) Ndopu. Accessed 26 April 2022. https://www.unsdgadvocates.org/edward‐ndopu

[2] LGBT Human Rights NASH SVIT Center . United against violence. LGBT+ situation in Ukraine in 2021. Accessed 26 April 2022. https://gay.org.ua/en/blog/2022/02/10/united‐against‐violence‐lgbt‐situation‐in‐ukraine‐in‐2021/

[3] ABC News . Thousands march in Ukraine for LGBT rights, safety. Accessed 26 April 2022. https://abcnews.go.com/International/wireStory/thousands‐march‐ukraine‐lgbt‐rights‐safety‐80107859

[4] The Washington Post . US letter to the UN alleging Russia is planning human rights abuses in Ukraine. Accessed 26 April 2022. https://www.washingtonpost.com/context/read‐u‐s‐letter‐to‐the‐u‐n‐alleging‐russia‐is‐planning‐human‐rights‐abuses‐in‐ukraine/93a8d6a1‐5b44‐4ae8‐89e5‐cd5d328dd150/?itid=lk_inline_manual_4

[5] Kottasová I. Eastern Europe was once a world leader on gay rights. Then it ran out of scapegoats. CNN. Accessed 26 April 2022. https://edition.cnn.com/2021/07/01/europe/lgbtq‐rights‐hungary‐eastern‐europe‐intl‐cmd/index.html

[6] Obangome GW , Bate F. Gabon votes to reverse ban on homosexuality. Reuters. Accessed 26 April 2022. https://www.reuters.com/article/us‐gabon‐lgbt‐lawmaking‐idUSKBN240258

[7] The Guardian . Botswana upholds ruling decriminalising same‐sex relationships. Accessed 26 April 2022. https://www.theguardian.com/global‐development/2021/nov/29/botswana‐upholds‐ruling‐decriminalising‐same‐sex‐relationships

[8] York G . For Africa's LGBTQ community, there's a glimmer of new hope amid prejudice. The Globe and Mail [Online]. Accessed 26 April 2022. https://www.theglobeandmail.com/world/article‐for‐africas‐lgbtq‐community‐theres‐a‐glimmer‐of‐new‐hope‐amid/

[9] Radio France Internationale . Gay couple in Namibia wins partial victory on residency application. Accessed 26 April 2022. https://www.rfi.fr/en/africa/20220307‐gay‐couple‐in‐namibia‐wins‐partial‐victory‐on‐residency‐application

[10] Frontline AIDS . Growing a movement: uniting the Kenyan LGBTQ community. Accessed 26 April 2022. https://frontlineaids.org/growing‐a‐movement‐uniting‐the‐kenyan‐lgbt‐community/

[11] Reid G . Angola decriminalizes same‐sex conduct. Human Rights Watch. Accessed 26 April 2022. https://www.hrw.org/news/2019/01/23/angola‐decriminalizes‐same‐sex‐conduct

[12] Reuters . Senegal rejects bid to toughen strict anti‐LGBT law. Accessed 26 April 2022. https://www.reuters.com/world/africa/senegal‐rejects‐bid‐toughen‐strict‐anti‐lgbt‐law‐2022‐01‐05/

[13] Cichowitz C , Rubenstein L , Beyrer C . Forced anal examinations to ascertain sexual orientation and sexual behavior: an abusive and medically unsound practice. PLoS Med. 2018;15(3):e1002536. 29547659 10.1371/journal.pmed.1002536 PMC5856262

[14] Deutsche Welle . Uganda introduces ‘Kill the Gays’ bill. Accessed 26 April 2022. https://www.dw.com/en/uganda‐introduces‐kill‐the‐gays‐bill/a‐50797504

[15] Human Rights Watch . “Tell Me Where I Can Be Safe”.

[16] Achimba CO . The new anti‐gay bill proposed in Ghana will destroy lives. Foreign Policy. Accessed 26 April 2022. https://foreignpolicy.com/2021/07/29/ghana‐nigeria‐africa‐lgbtq‐gay‐rights‐homosexuality‐crimnalized/

[17] Chen Y , Erdman J , Ezer T . Women living with HIV or AIDS & forced sterilization: a collaborative project of the Open Society Public Health Program and the University of Toronto Health Equity and Law Clinic. Accessed 26 April 2022. https://www.opensocietyfoundations.org/uploads/b06af101‐8e15‐42b8‐9891‐889036235e08/women‐living‐hiv‐aids‐forced‐sterilization‐20110701.pdf

